# Effect of Nebulized Verapamil on Oxygenation in Chronic Obstructive Pulmonary Disease (COPD) Patients Admitted to the Intensive Care Unit

**Published:** 2019-04

**Authors:** Guitti Pourdowlat, Reza Alizade Kashani, Fariba Ghorbani, Shadi Baniasadi, Hamidreza Jamaati, Behrooz Farzanegan

**Affiliations:** 1 Chronic Respiratory Diseases Research Center, National Research Institute of Tuberculosis and Lung Disease (NRITLD), Shahid Beheshti University of Medical Sciences, Tehran, Iran; 2 Tracheal Diseases Research Center, (NRITLD), Shahid Beheshti University of Medical Sciences, Tehran, Iran; 3 Critical Care Quality Improvement Research Center, Shahid Modarres Hospital, Department of Anesthesiology, Shahid Beheshti University of Medical Sciences, Tehran, Iran.

**Keywords:** Chronic Obstructive Pulmonary Disease, Calcium Channel Blockers, Verapamil, Oxygenation

## Abstract

**Background:**

Many pharmacological and behavioral therapies have been investigated to improve oxygenation in the intensive care unit (ICU). In patients with chronic obstructive pulmonary disease (COPD), the purpose of therapy is to correct the ventilation perfusion (V/Q) mismatch. Agents, such as calcium blockers, can affect both ventilation and vasculature. The inhalation route allows a more rapid achievement of therapeutic effects with few systemic side effects. Therefore, the present study aimed to investigate the effect of nebulized verapamil on oxygenation in COPD patients.

**Materials and Methods:**

In this double-blind, randomized clinical trial, twenty hypoxic COPD patients, admitted to ICU, were treated with 10 mg of verapamil twice daily for three days. Also, twenty patients with COPD, who were matched in terms of age, sex, and severity of the disease, were enrolled in the control group and received nebulized normal saline. The oxygenation parameters were compared using an arterial blood gas (ABG) test before and after the intervention.

**Results:**

The mean oxygen saturation was 91.2%±12.15 before verapamil inhalation, which increased to 95.75%±14.57 after receiving nebulized verapamil (P<0.05). Also, correction of blood pH, blood oxygen pressure, and oxygen ratio (PaO_2_/FIO_2_) were higher in patients receiving verapamil, compared to the control group. The length of hospital stay was similar in the two groups. During the first three days, 30% of patients in the verapamil group and 20% of patients in the control group were intubated.

**Conclusion:**

Our results indicated that verapamil inhalation increased oxygen saturation and accelerated extubation in patients with COPD.

## INTRODUCTION

According to the statistics published by the World Health Organization (WHO) in 2017, non-communicable diseases account for 40 million deaths per year; that is, 70% of total deaths worldwide (1). Following cardiovascular diseases and cancers, respiratory diseases are ranked the third most common diseases, followed by diabetes mellitus. Statistics show that 80% of Years Lived with Disability can be attributed to non-communicable diseases, which are classified into four categories of cardiovascular diseases, cancers, diabetes, and chronic respiratory diseases (2,3).

Chronic obstructive pulmonary disease (COPD), which is associated with a significant burden on the Iranian population, is a common disease, affecting more than 200 million people worldwide (4). Today, acute exacerbation of COPD and admission to the intensive care unit (ICU) are increasing due to urban pollution. In this regard, Varmaghani et al. (2016) reported the prevalence of COPD to be 9.2% in Tehran, Iran (5). Pulmonary symptoms in COPD are significantly heterogenic. As the disease progresses, deterioration of pulmonary gas exchange results in severe hypoxemia, with or without hypercapnia (6–8). In case of severe exacerbation, oxygen therapy and respiratory support are inevitable (9). Therefore, the main goal of treatment is to provide blood oxygenation and prevent its adverse effects.

To achieve the target saturation of 90±2% in patients at risk of hypercapnic respiratory failure, it is necessary to apply appropriate oxygen pressure and use a proper oxygen delivery device and medications for improving the oxygen uptake (10). Ventilation/perfusion (V/Q) mismatch is the main contributor to hypoxemia in COPD patients, resulting from remodeling and destruction of the pulmonary capillary bed, as well as progressive airflow limitation (11). Therefore, to improve oxygenation, correction of V/Q mismatch is a vital component of treatment.

To achieve the therapeutic goals in an ICU setting, several pharmaceutical and behavioral interventions have been investigated to improve oxygenation and reduce the length of hospital stay, especially in patients under mechanical ventilation. In this regard, semi-sitting and right/left lateral recumbent positions, as well as administration of nitric oxide and calcium channel blockers, have been investigated, and inconsistent results have been reported (10,12). Although the position did not significantly affect oxygenation, the nebulization of prostaglandins (PGI2) exerted significant effects on hypoxemia in the ICU setting (13). Also, the preventive effects of nebulized diltiazem on bronchospasm and irritation, caused by cold or exercise (14,15), have been reported. However, most studies on calcium channel blockers have focused on asthmatic patients, and the effects of medications as bronchodilators have been highlighted.

The efficacy of calcium channel blockers as pulmonary vasodilators is higher than their efficacy as airway dilators; therefore, their application as bronchodilators has been limited (16). Considering the effects of these agents, they are considered appropriate for the correction of V/Q mismatch in COPD patients. However, there is no study in the literature using calcium antagonists for the treatment of COPD patients.

Since the effects of calcium channel blockers depend on the type, dosage, and administration route (17, 18), in this study, we aimed to assess the efficacy of inhaled verapamil on oxygenation in COPD patients under intensive care.

## MATERIALS AND METHODS

### Study Design

This double-blind, placebo -controlled, randomized clinical trial was registered in the Iranian Registry of Clinical Trials (IRCT20170210032478N1). Also, the Research Committee of National Research Institute of Tuberculosis and Lung Diseases, affiliated to Shahid Beheshti University of Medical Sciences, approved this study.

Demographic and clinical information of the patients were collected using SPSS software and analyzed to determine the effect of verapamil inhalation on oxygenation from February 2018 to August 2018. The inclusion criterion was having COPD requiring ICU admission. On the other hand, the exclusion criteria were as follows: diagnosis of bradycardia; QT-interval disorders; atrioventricular block; arrhythmia; and an ejection fraction below 40% according to echocardiography. Also, patients were excluded if they used any β-adrenergic blockers or had a history of verapamil sensitivity.

### Measurements and Data Collection:

Considering the differences in the metabolic processes of systemic versus inhaled administration of verapamil (19), besides the safe dose of administration, we used 10 mg of verapamil (20) in a nebulizer at a concentration of 2.5 mg/mL, using an ultrasonic nebulizer (CUN60, CITIZEN^®^, Japan) at room temperature under close monitoring by the ICU physician (21). During this three-day study, the baseline measurements before verapamil or normal saline administration were compared with those after inhalation during three days of follow-up.

Also, in this double-blind, randomized clinical trial, the patients were assigned random codes to match the underlying variables, such as age and sex. After describing the objectives and processes of the study to the participants and receiving informed consent forms, Allen test was performed before radial artery cannulation to evaluate adequate collateral circulation to the palmar arches of the hand. First, the oxygenation status of all patients was evaluated using an ABG test by collecting 2 cc of arterial blood from the arterial line. The arterial line was placed at the radial artery insertion site after local anesthesia. With a catheter over the wire, the artery was punctured, and the cannula was inserted at a 45° angle. The catheter was fixed as soon as a flash of blood appeared in the needle.

Based on our previous pilot study, the sample size was considered to be 20 patients per group at 95% confidence interval and 80% power; the patients were enrolled in the study if they met the inclusion criteria. Next, the included patients were divided into two groups (intervention and control groups). The intervention group received 10 mg of verapamil, nebulized for three days (twice daily), whereas the control group received 2 cc of normal saline, nebulized for three days (twice daily).

Ten minutes before and after each inhalation dose, the ventilator parameter, that is, the fraction of inspired oxygen (FIO_2_), and O_2_ saturation were recorded and monitored. Also, an ABG test was performed to assess pH, partial pressure of oxygen (PaO_2_), and CO_2_ retention as the outcome variables. The ICU physician was informed about the interventions for ethical reasons so that he/she could make proper decisions if any adverse effects or suspicious conditions associated with verapamil inhalation were observed. The heart rate and blood pressure were monitored to assess safety; inhalation was terminated in the event of considerable changes.

### Statistical Analysis

To determine the patient’s response to verapamil, the results of ABG test before and after verapamil or normal saline administration were compared. Next, these comparisons were repeated with other doses and continued for three days. Repeated measures ANOVA test was also used to compare quantitative variables before and after treatment. The level of statistical significance was set at P<0.05 (two-sided). Statistical analysis was performed using SPSS Version 20 (SPSS, IBM, USA). Also, graphs were plotted using GraphPad Prism.

## RESULTS

All treatments were generally well-tolerated by the patients, and no adverse effects were reported; therefore, no patient discontinued the study. Twenty patients were included in the intervention group, and twenty patients were included in the control group. The mean age of the patients was 71.05±8.6 years in the intervention group and 68.25±7.4 years in the control group. The distribution of age in both groups was normal, based on the results of Kolmogorov–Smirnov test, and the student’s t-test showed no significant difference regarding age between the control and intervention groups. Also, the results showed that 45% of patients in the intervention group and 40% of patients in the control group were female and that there was no significant difference between the two groups in terms of gender.

**Figure 1.**
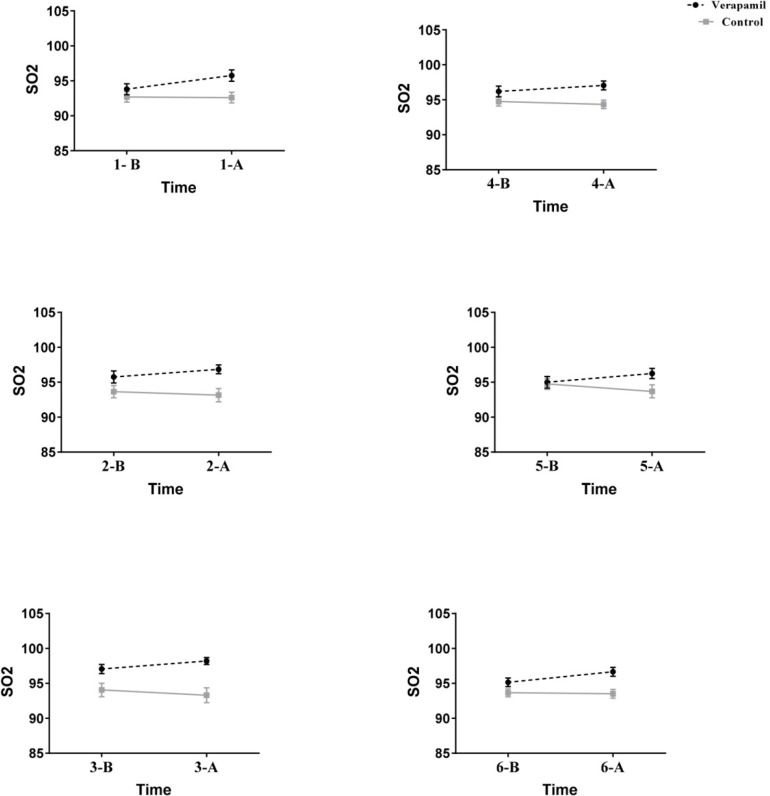
The modification obtained in SO2 among verapamil vs. control group during three days of follow up. O2 saturation significantly increased after verapamil inhalation in all three days. B: before; A: after, 1-B: before first administration; 1-A: after first administration; 2-B: before second administration; 2-A: after second administration; 3-B: before third administration; 3-A: after third administration.

The initial mean O_2_ saturation and pH were not significantly different in the two groups. As depicted in Figure 1, the mean O_2_ saturation was 87.2%±12.15 before verapamil administration, which increased to 95.75%±14.57 after receiving nebulized verapamil (P<0.05). On the other hand, there was no significant difference in the mean O_2_ saturation in the control group after receiving nebulized normal saline. Based on the results, the mean O_2_ saturation was 89.7±6.2% on the first day before normal saline administration, which increased to 90.6±3.4% after administration. There was no significant difference in terms of blood pH changes on the first and second days of verapamil administration, whereas, on the third day, the blood pH increased from 7.37±07.07 to 7.41±05.05. In the control group, no significant difference was observed regarding pH changes on three days of the trial.

**Figure 2.**
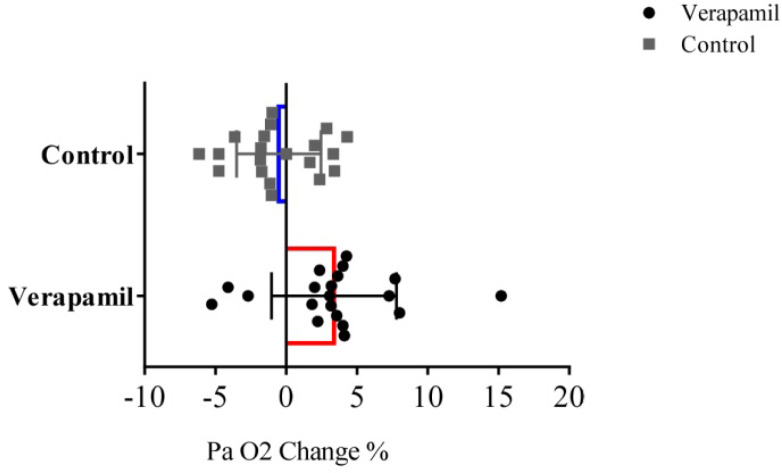
The amount of changes obtained in the both study groups. After three days Pa O2 significantly changed in the verapamil group

**Figure 3.**
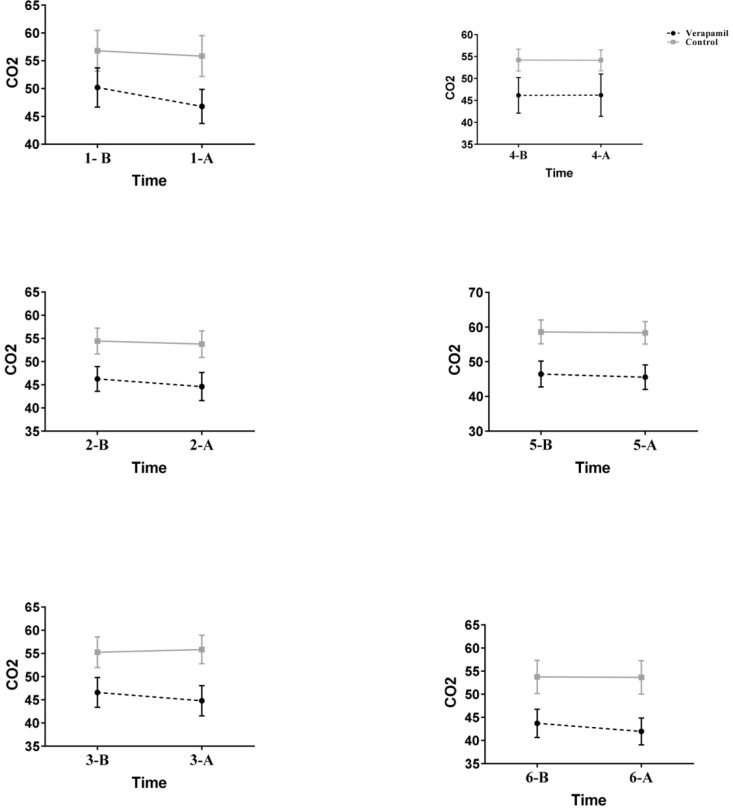
Changes rate in CO2 pressure among verapamil vs. saline patients. Base line PCO2 was not differing between groups. After the first verapamil inhalation PCO2 decreased significantly. B: before; A: after, 1-B: before first administration; 1-A: after first administration; 2-B: before second administration; 2-A: after second administration; 3-B: before third administration; 3-A: after third administration.

After the end of treatment, verapamil administration significantly increased the oxygen pressure from 76.35±15.05 to 110–130 mmHg. The change percentage was 3.37±0.98% in the verapamil group and −0.52±0.6% in the control group (P<0.05) (Figure 2). Moreover, CO_2_ pressure decreased from 56.8±6.51 to 41.9±5.15 mmHg; the change was significant on the third day after verapamil inhalation. However, in the control group, it decreased from 55.8 to 49.63, and no significant change was observed (Figure 3).

Moreover, changes in the blood bicarbonate were intangible. On the third day, the level of blood bicarbonate reached 28.9±3.4 mEq/L in patients receiving verapamil, and the difference with the control group was found to be significant. In the control group, no significant difference was found in bicarbonate correction. Also, the inspired oxygen concentration (PaO_2_/FiO_2_ ratio) was calculated in patients by normalizing oxygen pressure to inspiratory oxygen ratio to eliminate the effect of FIO_2_ and allow comparison of oxygenation among patients.

**Figure 4.**
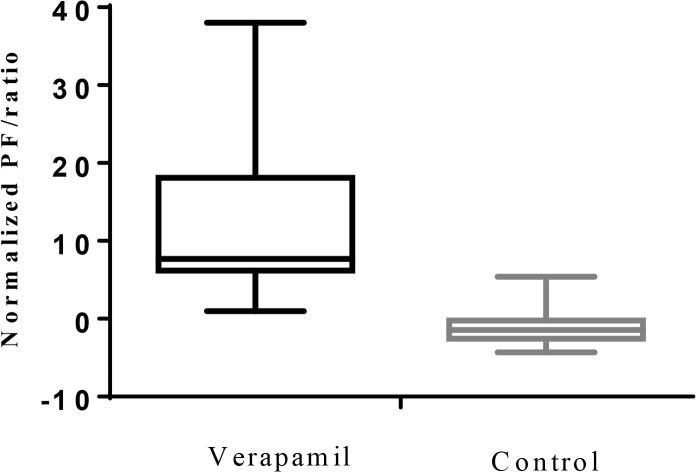
The average modification of O2 saturation based on normalization of FIO2 within three days of administration. PF ratio indicated significant increase in the patients received verapamil.

The PaO_2_/FiO_2_ ratio increased from 153.02±25.04 to 191.44±27.05 and 220±26.14 on the second and third days in the intervention group, respectively, and these changes were found to be significant. Conversely, in the control group, it increased from 125.69±32.12 to 160±35 and 153±25.72 on the second and third days, respectively, which was not significantly different during the three days of the follow-up. To compare the collected data, according to FIO_2_, the PaO_2_/FiO_2_ ratio is normalized in Figure 4. In the intervention group, the mean value of the normalized PaO_2_/FiO_2_ ratio was 12.24±2.3 versus −1±0.5 in the control group (P=0.05).

Regarding the safety of the intervention, it was found that 10 mg of inhaled verapamil did not affect the blood pressure or heart rate. The baseline blood pressure in the verapamil group was 92.7±10.8 mmHg, which reached 91.3±11.6 after the first administration. Also, the baseline heart rate was 95±20.3 bpm, which decreased to 93.4±17.2 bpm after the first intervention, without any significant difference. Also, no significant change was observed in the sequential administrations.

## DISCUSSION

In the present study, verapamil administration compensated for acidosis and increased oxygen pressure in ICU-admitted COPD patients. Considering the rapid onset of action following drug inhalation and low systemic absorption, verapamil was administered as an aerosol. In this study, the inhalation route of administration was safe, and no adverse effects were reported.

The correction of hypoxia in patients with respiratory failure is critical for the prevention of cascade complications. Since the clinical introduction of oxygen in 1783 for the treatment of hypoxic patients (23), numerous devices and methods have been employed to improve oxygenation. Overall, COPD patients experience respiratory acidosis during the acute phase of the disease, which may contribute to further tissue damage, progression of acute respiratory distress, and finally, ICU admission.

In our study, the ABG test demonstrated pH correction in the intervention group upon the first administration. After a period of three days, pH was similar in the intervention and control groups. However, the duration of intubation and length of hospital stay were not significantly different in the patients. Considering the effects of inhaled verapamil on the extubation time, maintenance of drug administration may lead to better oxygenation. In the present study, 30% and 20% of patients in the intervention and control groups were extubated within the first three days of admission, respectively (Table 1).

**Table 1.** Comparison of admission and extubation time among patients.

**Parameter**	**Verapamil**	**Normal Saline**	**P-value**
**Age (year)**	71.05 ± 8.6	68.25 ± 7.4	NS
**Gender (Female) %**	45	40	NS
**Duration of hospitalization**	8.2± 4.4	9.2± 5.1	NS
**Extubation within 3 days %**	30	20	0.04
**Mortality**	0	0	NS
**Re-intubation**	0	0	NS

Regarding the variety of contributing factors for hypoxemia in COPD, pulmonary hypertension may also impair oxygenation and be associated with a significant reduction in the patient’s survival (6). In this regard, various studies on the prevalence of pulmonary hypertension have yielded different results, ranging from less than 5% (24) to over 50% (25). It seems that underlying disorders, such as obstructive sleep apnea, thromboembolism, and heart failure, contribute to comorbidities (25).

Evidence shows that oxygen therapy improves the survival of hypoxic COPD patients through the prevention or reduction of pulmonary hypertension. However, few studies have investigated the role of calcium channel blockers in the improvement of oxygenation in COPD patients. Overall, aerosolized calcium blockers, due to their high concentration in the alveolar space (unlike their low systemic concentration), can dilate the pulmonary vasculature selectively, without affecting the systemic vasculature (22). Therefore, the inhalation of these agents may result in V/Q correction.

**Figure 5.**
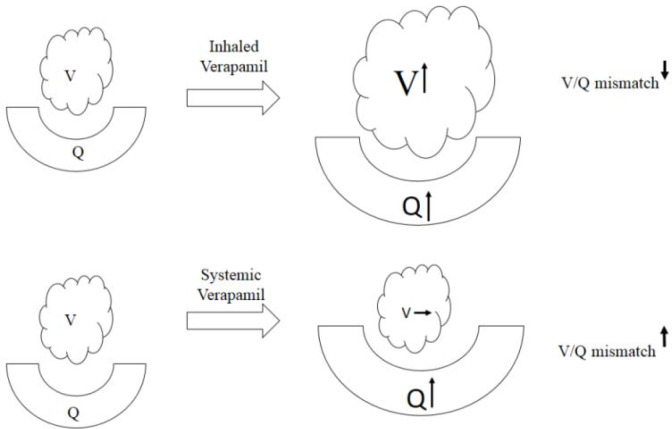
Effects of inhaled verapamil versus systemic verapamil. Aerosolized verapamil affects the both vascular as well as alveoli. Thus the well ventilated of well perfused regions result in correction of V/Q mismatch. While in systemic use, the impact of verapamil on vasculature network rather than pulmonary space would increase V/Q mismatch(22).

On the other hand, inhaled verapamil acts as a pulmonary vasodilator, but not a systemic vasodilator, particularly in the well-ventilated alveoli of the lung, receiving the highest dose of the aerosol agent (Figure 5). Accordingly, a previous study revealed a significant increase in O_2_ saturation and reported the improvement of function class, without considerable effects on pulmonary hypertension or FEV1. Unlike FEV1, FVC showed an increasing pattern; therefore, further ventilation of the dilated pulmonary vasculature might result in the correction of V/Q mismatch (26, 27).

Generally, the route of administration (e.g., oral, injection, and inhalation) can be a confounding variable (6). Some patients may develop bronchospasm due to the accumulation of calcium ions in the cytosol of airway smooth cells at the bronchial level (7). Also, the mechanism of action of verapamil in COPD patients differs from asthmatic patients. In COPD, the aerosolized use of this agent causes vascular dilatation in well-ventilated alveoli, and subsequently, decreases the V/Q mismatch. In this regard, Dernaika et al. reported an improvement in the V/Q ratio correction among patients with COPD due to an increase in gas exchange following iloprost inhalation therapy (28). They found that adequate ventilation with appropriate perfusion can correct the V/Q mismatch. Also, in an experimental study, the animals were treated with verapamil following stimulation with ovalbumin. The results indicated that the percentage of eosinophil cells, goblet cells, and mucosal production significantly reduced (29); all of these changes improved gas exchange in COPD patients.

This study had some limitations, including the unknown duration and stage of disease, and FEV1. Also, the correlation of smoking history, pulmonary artery pressure, and BMI with the improvement of oxygenation is another confounding factor. Finally, the inert gas test was not performed in this study to determine the effect of inhaled verapamil on the V/Q mismatch.

Based on the present results, owing to changes in PaO_2_ and hypercapnia, verapamil inhalation can be a suitable treatment option for COPD. Since some patients with COPD experience right heart failure syndrome or use other medications, such as digoxin and anticonvulsants, this treatment approach can be considered risk-free owing to the low systemic effects of inhalation.
